# Anti-inflammatory effects of rosuvastatin treatment on coronary artery ectasia patients of different age groups

**DOI:** 10.1186/s12872-020-01604-z

**Published:** 2020-07-11

**Authors:** Cheng-Hui Fan, Ying Hao, Yong-Hua Liu, Xiao-Lin Li, Zhen-Hao Huang, Yu Luo, Rui-Lin Li

**Affiliations:** 1 Department of Cardiology, Shanghai East Hospital, Tongji University, 1800 Yuntai Rd, Shanghai, 200126 People’s Republic of China; 2 Cardiovascular Medicine of Baoshan People’s Hospital of the Yunnan Province, Kunming Medical University, Baoshan, 678000 People’s Republic of China; 3 Department of Cardiology, Shanghai East Hospital (Ji’an Campus), Medical School, Jinggangshan University, Ji’an, 343009 People’s Republic of China

**Keywords:** Coronary artery ectasia, High-sensitivity C-reactive protein, Interleukin-6, Rosuvastatin

## Abstract

**Background:**

Coronary artery ectasia (CAE) is an angiographic finding of abnormal coronary dilatation. Inflammation plays a major role in all phases of atherosclerosis. We investigated the relationship between CAE and serum high-sensitivity C-reactive protein (hs-CRP) and interleukin-6 (IL-6) levels to test our hypothesis that patient age is associated with the efficacy of anti-inflammatory therapy for CAE.

**Methods:**

We conducted a prospective analysis of 217 patients with CAE treated at the Department of Cardiology, Shanghai East Hospital, Ji’an Campus and the Baoshan People’s Hospital, from January 1, 2015 to July 30, 2019. Baseline data of patients, including sex; age; and history of hypertension, hyperlipidemia, and diabetes, were collected from patient medical records. Study participants were grouped by age as follows: CAE-A (*n* = 60, age ≤ 50 years), CAE-B (*n* = 83, 50 years <age ≤ 70 years), and CAE-C (*n* = 74, age > 70). Additionally, there was a control (NC) group (*n* = 73) with normal coronary arteries.

**Results:**

All patients received oral rosuvastatin therapy (10 mg, QN quaque nocte) when they were diagnosed with CAE and maintained good follow-up, with a loss rate of 0.0% at the end of the 6-month follow-up. The NC group received regular symptom-relieving treatments and rosuvastatin therapy. Of these four groups, the inflammatory markers, hs-CRP and IL-6, were significantly higher in patients with CAE than in the NCs (*p* < 0.05). Post-hoc tests showed that hs-CRP and Il-6 levels had significant differences between the CAE-A and CAE-C groups (*P* = 0.048, *P* = 0.025). Logistic regression analysis showed that hs-CRP (OR = 1.782, 95% CI: 1.124–2.014, *P* = 0.021) and IL-6 (OR = 1.584, 95% CI: 1.112–1.986, *P* = 0.030) were independent predictors of CAE. The inflammatory markers were higher in the CAE-A group than in the CAE-B group and higher in the CAE-B group than in the CAE-C group. Follow-up after 6 months of rosuvastatin therapy showed a significantly greater reduction in hs-CRP and IL-6 levels in the CAE-A group than in the CAE-B group, which again were greater in the CAE-B group than in the CAE-C group.

**Conclusions:**

Anti-inflammatory therapy using rosuvastatin was more effective in younger CAE patients, indicating the need for early statin therapy in CAE.

## Background

Coronary angiography, a technique for diagnosing CAE, provides information on the size, location, and number of dilatations. CAE is characteristically defined as a dilated coronary artery segment whose diameter is at least 1.5 times that of the adjacent normal coronary lumen [1]. It is a relatively rare coronary artery abnormality (prevalence < 5%) among coronary artery diseases [2]. The increased prevalence of CAE in recent years has resulted in a greater focus on the incidence and factors influencing CAE; estimates of the prevalence of CAE have varied from 0.3 to 4.7% [3]. Nevertheless, etiopathogenetic mechanism of CAE is not yet completely known, although it may be related to systemic inflammation, stimulated nitric oxide production, coronary balloon angioplasty, extracellular matrix degradation, dyslipidemia, nodular polyarteritis, Kawasaki syndrome, and even genetic predisposition [4, 5]. CAE is more prevalent in patients with familial hypercholesterolemia (FH) than in those with coronary atherosclerosis and shows a strong inverse association with high-density lipoprotein cholesterol (HDL) cholesterol levels. This suggests that abnormal lipoprotein metabolism in individuals with FH predisposes patients to aneurysmal coronary artery disease [6]. Moreover, potential risk factors for CAE include an imbalance between matrix metalloproteinases (MMPs) and tissue inhibitor metalloproteinases (TIMPs), angiotensin-converting enzyme genotypes, a lower HDL cholesterol level, a higher low-density lipoprotein (LDL)/HDL ratio, elevated homocysteine levels, cocaine usage, smoking, vascular trauma, and diabetes [7–10]. Conventionally, CAE has been considered a variant of coronary atherosclerosis and an important clinical complication in interventional cardiology with increased thrombogenic potential of the ectatic arteries. CAE is closely related to myocardial infarction. However, there are currently no standard treatment guidelines specified for CAE. Anti-inflammatory and endothelium-protective effects of rosuvastatin have been suggested to improve the symptoms in patients with coronary artery disease [11]. Rosuvastatin is a selective hydroxy methylglutaryl coenzyme A (HMG-CoA) reductase inhibitor widely used for coronary atherosclerotic heart disease [12]. The liver is the main target organ of rosuvastatin, wherein it lowers cholesterol levels and increases the number of LDL receptors on the surface of liver cells, thereby improving lipid metabolism by promoting LDL absorption and inhibiting hepatic synthesis of very-low-density lipoprotein (VLDL) [13]. Statin therapy can exert pleiotropic effects in atherosclerotic processes, such as regulating inflammatory responses, endothelial function, and thrombus formation based on the reduction in LDL-C levels [14]. Rosuvastatin can also stabilize or reverse atherosclerotic plaques by suppressing MMP expression and protecting the vascular endothelium against inflammation [15, 16]. However, there is no conclusive evidence of therapeutic efficacy or optimal timepoint for rosuvastatin therapy in CAE patients in different age groups. We conducted this study to compare the inflammatory status and therapeutic effects of rosuvastatin in CAE patients in different age groups.

## Methods

### Study population

**Fig. 1:**
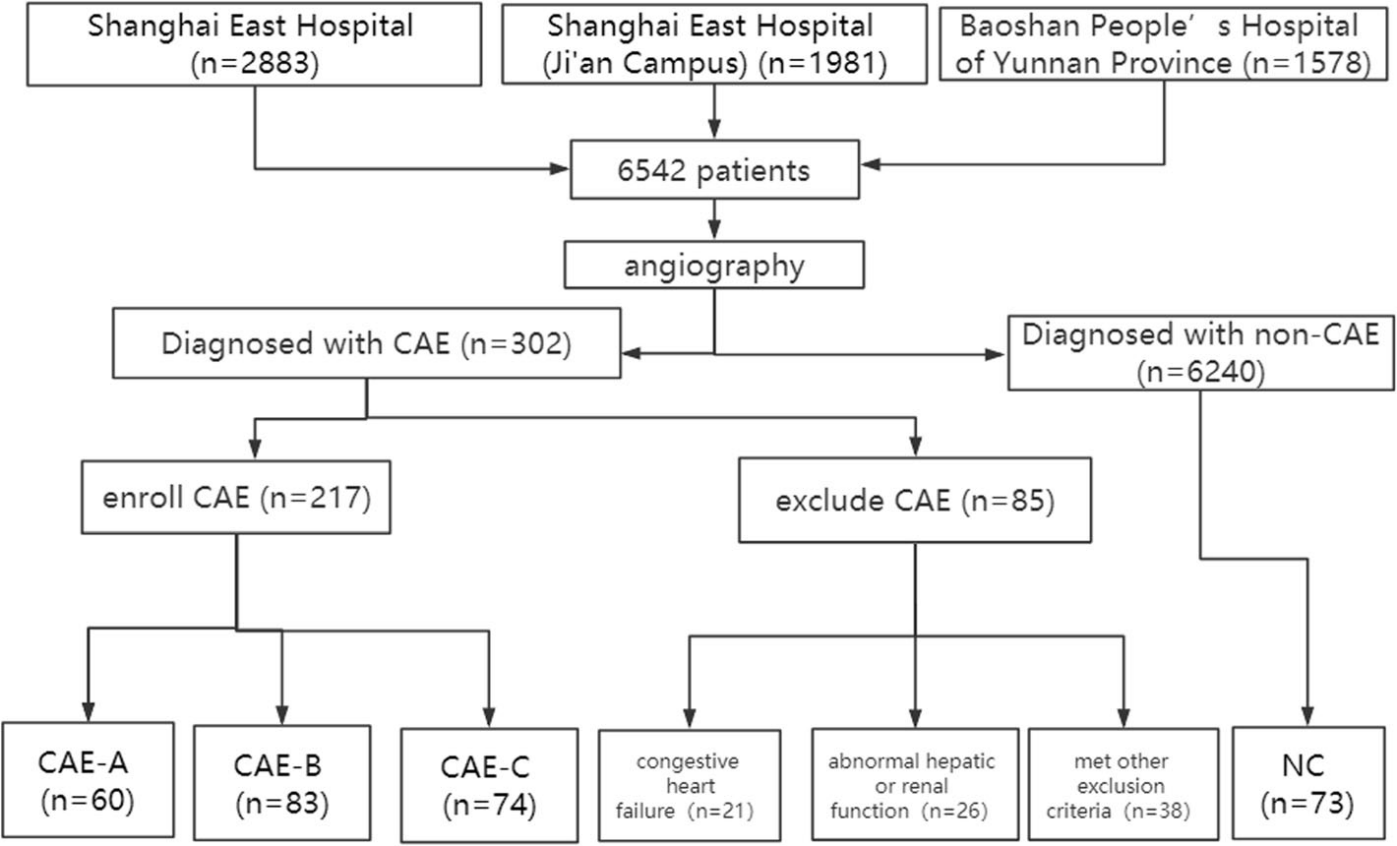
The patient screening flow chart

We prospectively enrolled 6542 patients who were first diagnosed using coronary angiography at our centers from January 1, 2015, to July 30, 2019. The exclusion criteria included various malignant tumors, intolerance to statin treatment, dilated segments appearing within or directly associated with coronary bypass grafts, coronary dilation development after coronary interventions, a diagnosis of Kawasaki disease, previous statin treatment, or coronary artery anomalies, acute or chronic coronary total occlusion, acute coronary syndrome [17], or inability to complete a 6-month follow-up. Finally, 302 patients were diagnosed with CAE, and 85 patients were excluded (Fig. 1). We also included 73 normal controls. Of the excluded patients, there were 21 patients with congestive heart failure, 26 with abnormal hepatic or renal function, and 38 who met other exclusion criteria. We followed up a total of 290 patients and divided them into four groups: normal control (NC; *n* = 73), CAE-A (*n* = 60), CAE-B (*n* = 83), and CAE-C (*n* = 74) (Fig. 1). Samples were collected for clinical data, including blood lipids, high-sensitivity C-reactive protein (hs-CRP), interleukin-6 (IL-6) and other biochemical indicators (Table 1). The patient and control groups received rosuvastatin treatment upon CAE diagnosis, not oral statin therapy before the time of enrollment.

**Table 1:** Basic information and laboratory findings of CAE patients and normal controls

Groups	NC group (*n* = 73)	CAE-A (*n* = 60) (age ≤ 50 years)	CAE-B (*n* = 83) (50 years<age ≤ 70 years)	CAE-C (*n* = 74) (age > 70 years)	Total CAE (A + B + C, *n* = 217)	*p*-value	CAE-A vs CAE-B	CAE-B vs CAE-C	CAE-A vs CAE-C
Sex (M), n (%)	42 (57.5)	39 (65.0)	57 (68.7)	50 (67.6)	146 (67.3)	0.2794			
Diabetes mellitus, n (%)	13 (17.8)	11 (18.3)	18 (21.6)	18 (24.3)	47 (21.7)	0.1712			
Hypertension, n (%)	23 (31.5)	19 (31.7)	28 (33.7)	27 (36.5)	74 (34.1)	0.2142			
Waist circumference (cm)	90.3 ± 14.8	91.6 ± 12.7	92.7 ± 16.1	93.8 ± 19.1	92.8 ± 16.2	0.5277			
Creatinine (mg/dL)	70.34 ± 11.6	67.6 ± 12.7	77.8 ± 13.2	87.8 ± 14.8	78.48 ± 12.5	0.2560			
Smoking index	119.2 ± 15.5	110.3 ± 10.5	135.5 ± 15.6	130.2 ± 15.1	126.7 ± 17.8	0.2391			
Total cholesterol (mmol/L)	4.89 ± 1.04	5.59 ± 1.21^*^	5.39 ± 1.18	4.95 ± 1.07	5.30 ± 1.16^*^	0.0452	0.321	0.252	0.175
Low-density lipoprotein-C (mmol/L)	2.89 ± 0.36	3.92 ± 0.54^*^	3.74 ± 0.51^*^	3.73 ± 0.49^*^	3.79 ± 0.52^*^	0.0237	0.434	0.523	0.259
High-density lipoprotein-C (mmol/L)	1.08 ± 0.12	1.22 ± 0.27	1.15 ± 0.19	1.13 ± 0.15	1.16 ± 0.16	0.9161			
Triglycerides (mmol/L)	1.56 ± 0.19	1.69 ± 0.19	1.89 ± 0.21	1.75 ± 0.18	1.78 ± 0.15	0.8493			
High-sensitivity CRP (mg/L)	16.9 ± 3.82	32.3 ± 5.51^*^	26.1 ± 4.23^*^	22.5 ± 4.82^*^	25.6 ± 4.65^*^	0.0213	0.230	0.198	0.048^*^
Glycated hemoglobin (%)	5.89 ± 1.12	5.90 ± 1.07	6.50 ± 1.12	6.89 ± 1.25	6.46 ± 1.21	0.1421			
Ejection fraction (%)	56.3 ± 12.58	59.4 ± 9.14	55.5 ± 9.23	50.3 ± 8.47	54.8 ± 8.53	0.3432			
Hemoglobin (g/L)	125.5 ± 26.5	131.1 ± 28.6	125.1 ± 26.3	119.1 ± 29.8	123.8 ± 28.1	0.6535			
Red blood cell distribution width (%)	36.8 ± 5.26	36.7 ± 5.95	38.7 ± 4.94	36.4 ± 4.68	37.4 ± 5.59	0.8601			
Mean platelet volume (fL)	10.5 ± 1.26	10.7 ± 1.05	11.2 ± 1.35	10.5 ± 1.91	10.8 ± 1.45	0.2503			
WBC (10^9^/L)	6.25 ± 2.56	8.85 ± 2.21	8.35 ± 2.06	8.25 ± 2.36	8.38 ± 2.30	0.0763			
Neutrophils (10^9^/L)	4.12 ± 1.69	6.12 ± 1.58	5.76 ± 1.49	5.66 ± 1.52	5.89 ± 1.60	0.0752			
Lymphocytes (10^9^/L)	0.50 ± 0.22	0.65 ± 0.25	0.68 ± 0.23	0.72 ± 0.20	0.70 ± 0.23	0.3854			
Neutrophils/Lymphocytes ratio	8.18 ± 5.36	9.42 ± 6.89	8.48 ± 6.56	7.87 ± 6.36	8.41 ± 6.60	0.1025			
Interleukin-6 (pg/dL)	4.1 ± 0.6	12.3 ± 1.5^*^	10.9 ± 1.3^*^	8.9 ± 1.1^*^	10.6 ± 1.3^*^	0.001	0.120	0.095	0.025^*^

*:NC group vs. Total CAE group. *p*-value is considered significant if < 0.05

The primary endpoint included confirming the presence of inflammatory markers such as hs-CRP and IL-6 after 6 months of treatment. The secondary endpoint included measuring hs-CRP and IL-6 levels and observing any side effects of rosuvastatin. According to the results of coronary angiography and patients’ ages, participants were divided into four groups: CAE-A (age ≤ 50 years), CAE-B (50 years <age ≤ 70 years), CAE-C (age > 70 years), and NC (age-matched) with normal coronary arteries. According to the 1:1 age-matching, other risk factors (such as sex, hypertension, and diabetes) were matched for as much as possible, except for the CAE status. This study was approved by the medical ethics committee of the Shanghai East Hospital, Shanghai East Hospital (Ji’an Campus), and Baoshan People’s Hospital of the Yunnan Province.

### Measurement of related indicator characteristics

Hypertension was defined as a systolic blood pressure (SBP) ≥140 mmHg and/or diastolic blood pressure (DBP) ≥90 mmHg [18]; BP was measured three times on the same day for patients not taking antihypertensive drugs. The smoking index was calculated as the number of cigarettes smoked per day × the number of years of smoking [19]. Fasting venous blood samples of all subjects were collected to measure hematological parameters and biochemical indices. The red blood cell distribution width, hemoglobin level, mean platelet volume, and white blood cell (WBC) counts were analyzed using a Horiba ABX 80 Diagnostics (ABX pentra Montpellier, France). Serum glucose and creatinine, HDL-C, LDL-C, total cholesterol (TC), triglycerides (TGs), hs-CRP, and interleukin-6 (IL-6) levels were detected with enzymatic colorimetric methods using a fully automatic biochemical analyzer (Roche Cobas c702) in Shanghai East Hospital, Shanghai East Hospital (Ji’an Campus), and Baoshan People’s Hospital of the Yunnan Province.

### Coronary angiography

**Fig. 2:**
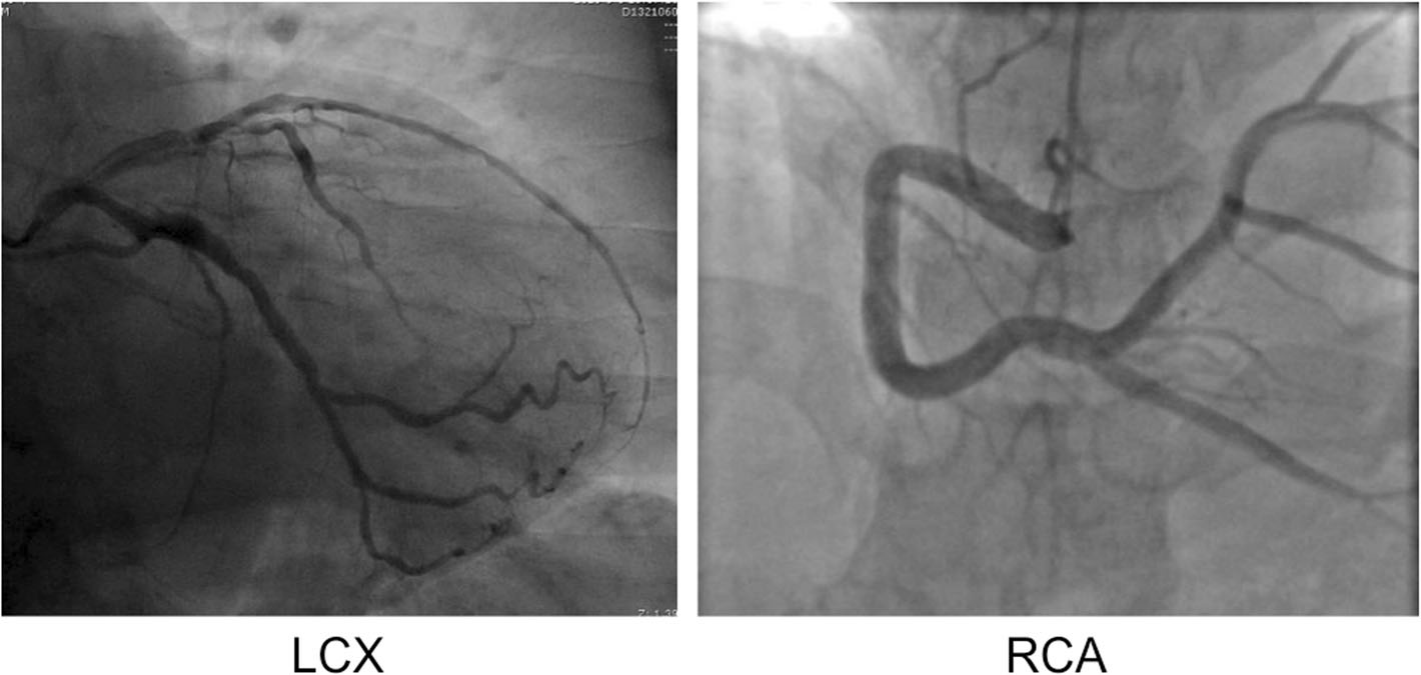
Representative coronary artery ectasia images of left circumflex branch (LCX) and right coronary artery (RCA)

The Siemens Artis zeego III was used to conduct coronary angiography with a routine radial artery approach. X-ray photography was performed with the injection of a contrast agent. Blood vessel diameter measurements were performed by skilled coronary intervention doctors. CAE was defined as local or diffuse dilated coronary arteries with diameters exceeding 1.5 fold that of the adjacent normal coronary lumen [20]. The coronary artery images considered indicative of CAE after qualitative comparative analysis by two independent operators were included in this study. Representative CAE images of the left circumflex branch (LCX) and right coronary artery (RCA) are shown (Fig. 2). Pharmacological therapy was withheld for at least 24 h before angiography.

### Statistical analysis

Statistical analysis was conducted using SPSS software version 19.0. Continuous variables are presented as means ± standard deviations for normally distributed data, while non-normally distributed data are presented as medians. Comparisons between two groups were conducted using an independent samples *t*-test and qualitative data were evaluated by Fisher’s exact test. A comparison of continuous variables between the three groups was performed using the one-way ANOVA with post hoc Dunnett’s correction. A *p*-value < 0.05 was considered statistically significant. Logistic regression analysis and stepwise methods were applied to screen the factors showing correlations with CAE, with the entry criterion set at *p* < 0.05 and the rejection criterion at *p* > 0.05.

## Results

From the study cohort, 302 patients were diagnosed with CAE, of which 85 were excluded based on the exclusion criteria. In addition, 73 patients were used as control group (NC group). The prevalence of CAE among all patients who underwent angiography in this study was 302/6542 = 0.046, representing a 4.6% prevalence among all patients undergoing angiography.

Baseline characteristics of risk factors for CAE, including sex (male), hypertension, diabetes, hyperlipidemia, and smoking history, were similar in the total CAE and NC groups (Table 1). Particularly, the WBC counts were not significantly different between the groups. In addition, subclasses of leukocytes, such as neutrophils and lymphocytes, were not significantly different between the groups.

Laboratory findings such as TC, LDL-C, hs-CRP, and IL-6 were significantly higher in the total CAE group than in the NC group (*p* < 0.05). TC, LDL-C, hs-CRP, and IL-6 levels were significantly higher in the CAE-A group than in the CAE-B, CAE-C, and NC groups; furthermore, TC, LDL-C, hs-CRP, and IL-6 levels were significantly higher in the CAE-B group than in the CAE-C or NC group. Under similar circumstances, TC, LDL-C, hs-CRP, and IL-6 levels were significantly higher in the CAE-C group than in the NC group. There were significant differences in hs-CRP (*P* = 0.048) and IL-6 (*P* = 0.025) levels but not in TC (*P* = 0.175) and LDL-C (*P* = 0.259) levels between the CAE-A and CAE-C groups. There were no significant differences in sex, hypertension, diabetes, waist circumference, smoking index, TGs, glycosylated hemoglobin, red blood cell distribution width (RBW), or mean platelet volume among the three different age groups of CAE patients (*p* > 0.05; Table 1).

Logistic regression analysis was performed to identify independent risk factors associated with CAE. In the stepwise analysis, covariant factors included hypertension, diabetes mellitus, hs-CRP, LDL-C, smoking, triglycerides, WBC, and IL-6. Multivariate analysis showed that increased hs-CRP and IL-6 levels were independent predictors of CAE (Table 2), whereas multivariate analysis revealed only increased hs-CRP (OR, 1.782; CI, 1.124–2.014; *p* = 0.021) and IL-6 (OR, 1.584; CI, 1.112–1.986; *p* = 0.030) levels as independent predictors (Table 2). Baseline drug treatments of patients with confirmed diagnoses of CAE at study inclusion are shown in Table 3. No significant differences were observed in the baseline medication among the CAE groups (Table 3). There were no significant differences in the selection of therapeutic medications between the three CAE groups and the NC group (*p* > 0.05). As far as compliance issues are concerned, we were dedicated to ensure regular health education and regular outpatient follow-up visits. We also kept reminding patients to take medication by telephone, WeChat, and other means. For various reasons such as financial constraints, some patients could not access drugs.

**Table 2:** Multivariate analysis of variables associated with CAE

	OR	95% CI	*p*-value
Hypertension	1.364	0.932–1.648	0.248
Diabetes mellitus	1.407	0.802–2.053	0.198
Waist circumference	1.448	0.967–1.938	0.124
Total Cholesterol	1.462	0.970–1.965	0.112
LDL-C	1.492	0.986–2.091	0.099
Smoking	1.119	0.932–1.422	0.176
TGs	1.238	0.836–1.865	0.236
WBC	1.690	0.990–1.785	0.061
hs-CRP	1.782	1.124–2.014	0.021^*^
Interleukin-6	1.584	1.112–1.986	0.030^*^

*CAE* coronary artery ectasia, *hs-CRP* high-sensitivity C-reactive protein, *LDL-C* low-density lipoprotein cholesterol, *TGs* triglycerides, *WBC* white blood cells. *: *p* < 0.05 vs NC group OR, odds ratio and CI, confidence interval

**Table 3:** Baseline medication selection of CAE patients after confirmed diagnosis

Treatments	Group NC (*n* = 73)	CAE-A (*n* = 60) (age ≤ 50 years)	CAE-B (*n* = 83) (50 years<age ≤ 70 years)	CAE-C (*n* = 74) (age > 70 years)	Total CAE (A + B + C, *n* = 217)	*p*-value
ACEI/ARB	18	16	27	28	71	0.895
β-receptor blocker	21	25	36	28	89	0.424
Rosuvastatin	73	60	83	74	217	0.945
Calcium-channel blocker	16	18	15	17	50	0.310
Diuretics	14	15	13	16	44	0.769
Aspirin	35	42	56	50	148	0.424

*p*-value: total CAE vs NC. *CAE* coronary artery ectasia, *ACE* angiotensin-converting enzyme

After the 6-month treatment with rosuvastatin, serum hs-CRP and IL-6 levels were reduced in the three CAE age groups (Table 4), supporting the efficacy of rosuvastatin as an anti-inflammatory agent. Of the three CAE age groups, the CAE-A (age ≤ 50 years) group showed the greatest effect of rosuvastatin treatment, as evidenced by the most significant reduction in serum hs-CRP (delta value was 15.1 ± 3.33, *P* = 0.0001) and IL-6 (delta value was 5.9 ± 1.6, *P* = 0.021) levels. This group showed the greatest reduction in serum hs-CRP and IL-6 levels, followed by the CAE-B group (delta value of hs-CRP was 9.4 ± 2.86, *P* = 0.023, delta value of IL-6 was 3.0 ± 1.5, *P* = 0.043). The results of the follow-up found that younger patients had a greater reduction in serum hs-CRP and IL-6 levels, suggesting that rosuvastatin had a greater anti-inflammatory effect in younger patients (Table 4).

**Table 4:** Comparison of serum hs-CRP and IL-6 levels in CAE patients treated with rosuvastatin

Groups	CAE-A (*n* = 60) (age ≤ 50)				CAE-B (*n* = 83) (50 < age ≤ 70)				CAE-C (*n =* 74) (age > 70)			
	Pretreatment	Posttreatment	*Delta*	*p*-value	Pretreatment	Posttreatment	*Delta*	*p*-value	Pretreatment	Posttreatment	*Delta*	*p*-value
hs-CRP (mg/L)	32.3 ± 5.51	17.5 ± 2.38 ^*^	15.1 ± 3.3	0.0001	26.1 ± 4.23	18.8 ± 2.74 ^*^	9.4 ± 2.86	0.023	22.5 ± 4.82	19.8 ± 2.98	4.5 ± 3.12	0.310
IL-6 (pg/dL)	12.3 ± 1.5	6.4 ± 1.7^*^	5.9 ± 1.6	0.021	10.9 ± 1.3	7.5 ± 2.0^*^	3.0 ± 1.5	0.043	8.9 ± 1.1	7.6 ± 2.3	1.9 ± 1.5	0.519

*CAE* coronary artery ectasia, *hs-CRP* high-sensitivity C-reactive protein, *IL-6* interleukin-6. Pretreatment: values measured at study inclusion. Posttreatment: values measured after 6 months of treatment with rosuvastatin. *: *p* < 0.05 vs corresponding pretreatment group

## Discussion

Dyslipidemia is a well-recognized, major risk factor for atherosclerosis [21, 22]. Increased serum lipids, especially LDL-C, can deposit in the arterial wall and gradually form atherosclerotic plaques, which can consequently block the native artery and cause cardiovascular diseases, such as coronary heart disease [23]. Increased inflammation is the core process in all stages of atherosclerosis. With the application and development of several techniques such as anti-inflammatory therapy, antithrombotics, thrombolysis drugs, and catheter treatment in recent decades, the incidence and mortality due to atherosclerosis or obstructive vascular diseases have been significantly reduced [24, 25]. CAE is a multifactorial disease, and its pathogenic mechanism has not yet been fully elucidated. CAE is considered a variation of atherosclerosis, mainly resulting from the thinning and/or destruction of the myocardial membrane. However, the dilatation process may be independent of the atherosclerotic process because it can be found as an isolated lesion in the coronary arteries and other vascular systems [26]. Dahhan A argued that atherosclerotic CAE does not carry additional risks compared to atherosclerotic coronary artery disease without ectasia [27]. Aksu T, Uygur B, Kosar, suggested that risk factors for CAD and the clinical presentation of CAE were considerably similar, and this situation was consistent with the similar etiopathogenesis of the two diseases [28]. Elevated inflammatory markers, such as plasma IL-6 and plasma soluble adhesion molecules, are closely linked to the presence of coronary artery dilation [29–31]. One study has indicated increasing evidence that neutrophils and neutrophil-derived products participate in atherogenesis and CAE [32]. The neutrophil/lymphocyte ratio was higher in patients with CAD, coronary slow flow, and CAE than in those with a normal coronary anatomy. The NLR may be an indicator of CAD, CAE, and coronary slow flow. This finding suggests that a more severe inflammatory process could be involved in CAE development [33–35]. In our study, we also studied the NLR between the NC and total CAE groups; the NLR was higher in the total CAE group than in the NC group, indicating the tendency of severe inflammation in CAE, although the result was not statistically significant (*P* = 0.1025). A more significant level of chronic inflammation might be linked with CAE pathogenesis, which is associated with both inflammatory markers and inflammatory cells in CAE patients [36].

Long-term exposure to nitrites, herbicide sprays, acetylcholine inhibitors, cocaine, and smoking can also lead to degeneration of the endothelium of the coronary arteries through oxidative stress-induced inflammation, which can eventually cause CAE [12]. Research on inflammation and CAE has characterized CAE-related inflammation, which includes elevated hs-CRP and IL-6 levels [37]. Accumulation of excess circulating LDL-C was associated with an overproduction of reactive oxygen species and increase in proinflammatory cytokines in the coronary endothelium, linking elevated cholesterol with cardiovascular inflammation [38].

Limited studies have focused on the inflammatory status of different age groups of CAE patients. TC and LDL-C levels are important for risk evaluation of coronary heart disease, which benefits from statin therapy through the reduction in LDL-C, hs-CRP, and IL-6 levels [39]. We hypothesized that increased inflammatory marker levels observed in younger patients (CAE-A) can be explained by several factors. First, younger patients are more likely to be stressed, resulting in a more primed or activated inflammatory status [40]. In addition, younger patients responded more strongly to physical and emotional stimulation, which can lead to increased inflammatory marker levels. There are other lifestyle factors that can also lead to inflammation, such as cocaine abuse and trauma [41].

Previous prospective studies have also found that statins can efficiently slow down the growth rate of an abdominal aortic aneurysm compared with the growth rate of abdominal aortic aneurysms in controls [42]. In the present study, the efficacy of rosuvastatin in CAE patients in different age groups was investigated and compared. The findings may be explained by higher inflammatory marker levels in younger patients than in older patients; thus, the same dose of rosuvastatin could be more likely to produce a greater anti-inflammatory effect. Moreover, a smaller percentage of younger people had never taken rosuvastatin before. Older patients had a higher proportion of rosuvastatin history because of arteriosclerosis, hyperlipidemia, and stroke, among other health complications. Therefore, the lipid-lowering effect of rosuvastatin may be more potent, which boosts its anti-inflammatory effects in young patients. The Cholesterol Treatment Trialists’ Collaboration reported that the efficacy of statin therapy was lower in older patients than in younger patients [43]. Furthermore, younger individuals have a higher basal metabolism level with regard to lipid synthesis and degradation [44]; therefore, younger CAE patients could be more sensitive to rosuvastatin treatment. After rosuvastatin treatment, hs-CRP and IL-6 levels of the CAE-A group were reduced to levels comparable to those of the NC group, while those of the CAE-C group were only partially reversed, indicating that the inflammatory status of younger CAE patients was more severe but reversible, while that of older CAE patients was comparatively mild, persistent, and irreversible.

### Study limitations

First, this study was based on a relatively small number of patients, although a large sample size was examined. Second, although specific exclusion criteria were chosen, some confounding factors may still have caused interference, for example, the accurate assessment of coronary artery diameter may have been limited due to uncertainty in identifying the reference parts of the vessel. It would have been better to use intravascular ultrasound or optical coherence tomography to provide more accurate information about the vessel. Third, pharmacological therapy was withheld for at least 24 h before cardiac catheterization, but this period may not have been a long enough washout period to exclude possible effects of drugs on the plasma inflammatory markers.

## Conclusions

Younger CAE patients had higher inflammatory marker levels than older CAE patients. The greatest efficacy of anti-inflammatory treatment was found in younger CAE patients, suggesting that rosuvastatin should be prescribed at the time of CAE diagnosis, especially in younger patients.

## Data Availability

Study protocol and data set: Not available. Statistical code: Available from Dr. Luo (e-mail, luoyu201909@163.com).

## Abbreviations

ACEIs: Angiotensin-converting enzyme inhibitors
ARBs: Angiotensin-receptor blocker
CAE: Coronary artery ectasia
hs-CRP: High-sensitivity C-reactive protein
IL-6: Interleukin-6
LDL-C: Low-density lipoprotein cholesterol
PO: Per os
QN: Quaque nocte
WBC: White blood cell
